# M_x_C_y_-Type Nanocarbide Crystallization in CrMnFeCoNi, CrMnFeCoNiV_0.5_, and CrMnFeCoNiMo_0.5_ HEAs Manufactured Through Powder Metallurgy

**DOI:** 10.3390/nano16100592

**Published:** 2026-05-12

**Authors:** Alfredo Martinez Garcia, Carlos Gamaliel Garay Reyes, Erick Adrián Juárez Arellano, Marco Antonio Ruiz Esparza Rodríguez, José Manuel Mendoza Duarte, Irving Ignacio López López, Juan Carlos Guía Tello, Gustavo Rodríguez Cabriales, Sergio González, Emmanuel José Gutiérrez Castañeda, Roberto Martínez Sánchez

**Affiliations:** 1 Departamento de Metalurgia e Integridad Estructural, Centro de Investigación en Materiales Avanzados, Avenida Miguel de Cervantes Saavedra 120, Chihuahua C.P. 31136, Mexico; alfredo.martinez@cimav.edu.mx (A.M.G.); marco.ruiz@cimav.edu.mx (M.A.R.E.R.); irving.lopez@cimav.edu.mx (I.I.L.L.); 2 Centro de Investigaciones Científicas, Instituto de Química Aplicada, Campus Tuxtepec, Universidad del Papaloapan, Circuito Central 200, Col. Parque Industrial, Tuxtepec C.P. 68301, Mexico; eajuarez@unpa.edu.mx; 3 Departamento de Física de Materiales, Centro de Investigación en Materiales Avanzados, Avenida Miguel de Cervantes Saavedra 120, Chihuahua C.P. 31136, Mexico; jose.mendoza@cimav.edu.mx; 4 Departamento Metal Mecánica, Tecnológico Nacional de Mexico-Instituto Tecnológico de Saltillo, Blvd. Venustiano Carranza 2400, Saltillo C.P. 25280, Mexico; juanc.gt@saltillo.tecnm.mx; 5 Departamento de Metalmecánica, Tecnológico Nacional de Mexico-Instituto Tecnológico de Chihuahua, Avenida Tecnológico 2909, Chihuahua C.P. 31200, Mexico; gustavo.rc@chihuahua.tecnm.mx; 6 Materials Science and Engineering Department, Alvaro Alonso Barba Technological Institute, Universidad Carlos III de Madrid, Avenida Universidad, 28911 Leganés, Spain; sgonsan@ing.uc3m.es; 7 Instituto de Metalurgia, Universidad Autónoma de San Luis Potosí, Sierra Leona No. 550, San Luis Potosí C.P. 78210, Mexico; emmanuel.gutierrez@uaslp.mx

**Keywords:** nanocarbides crystallization, HEAs, thermodynamic parameter, DTA analysis

## Abstract

The study presents a comprehensive report on the kinetic and thermodynamic parameters of carbide crystallization in CrMnFeCoNi, CrMnFeCoNiV_0.5_, and CrMnFeCoNiMo_0.5_ HEAs. This study considers only carbon resulting from the decomposition of the process control agent, which diffuses and becomes trapped within the HEA structure (0.79–0.91 wt.% C). The crystallization and growth of the carbides were monitored through thermal analysis and thermo-XRD at different temperatures. The activation energy was calculated using the Kissinger and Flynn/Wall/Ozawa methods, and the crystallization kinetics were evaluated using the Avrami–Erofeev model. The results of the XRD analyses and DTA curves of the CrMnFeCoNi, CrMnFeCoNiV_0.5_, and CrMnFeCoNiMo_0.5_ HEAs showed the following nanocarbide crystallization sequences: M_7_C_3_→MC→M_3_C_2_, M_23_C_6_, and MC→M_6_C, respectively. The transitions observed in the DTA curves were associated with M_7_C_3_, M_23_C_6_, and MC phases with activation energies (*E_a_*) of 238–251, 188–203, and 326–341 kJ/mol, respectively. Furthermore, kinetic analyses indicate that the crystallization of M_x_C_y_-type carbides occurs via nucleation.

## 1. Introduction

New technological requirements demand materials with enhanced properties compared to traditional alloys, such as steels and superalloys. Consequently, a novel category of alloys has emerged from these requirements, designated as high-entropy alloys (HEAs) [1,2]. These alloys exhibit excellent mechanical and functional properties [3]. For this reason, the analyses and investigations of methods for the large-scale production of HEAs are important. Among them, powder metallurgy is one of the most widely used manufacturing routes for forming HEAs [4]. However, mechanical alloying through high-energy ball milling (HEBM) commonly uses carbon-rich process control agents (PCAs), such as stearic acid, isopropyl alcohol, ethanol, n-heptane, and toluene, to prevent particle agglomeration [5]. During the milling process, this carbon gets generally trapped within the interstices and structural defects of HEAs and during a subsequent heat treatment, it often reacts with the metallic matrix to form carbides (M_x_C_y_) [6,7]. The crystallization of carbides in the HEA matrix improves its structural properties for applications under severe conditions, as seen in Fe3C-reinforced steels or WC cermets [8,9]. Therefore, a HEA matrix with carbide can be classified as a refractory high-entropy alloy (RHEA). However, other manufacturing routes include hot pressing, electric arc melting, high-frequency induction melting, spark plasma sintering, and sputtering deposition [10]. These methods prevent carbide crystallization and reduce the time required for subsequent heat treatment [11]. In addition, all of them require specialized operating conditions and specialized equipment, which are not widely used for producing alloys on an industrial scale [11].

The presence of refractory elements, such as Cr, V, and Mo, has been shown to enhance the mechanical properties of CrMnFeNiCo-based HEAs produced by mechanical alloying. However, a previous study observed that CrMnFeNiCo-based HEAs form carbides rich in refractory elements after a heat treatment [12]. Hence, the study of carbide crystallization during thermal process of HEAs manufactured through powder metallurgy is an emerging field of research. In addition, there is a great need to study the thermal stability of the structure and microstructure of HEAs manufactured through HEBM. Although theoretical studies have addressed the kinetics of carbide precipitation in HEAs [13], further experimental research is needed to advance our understanding in this field. It is essential to examine the fundamental thermodynamic parameters governing carbide precipitation in HEAs, including reaction enthalpy (*ΔH_r_*) and activation energy (*E_a_*). Therefore, this study evaluates the influence of refractory elements, such as V and Mo, on the microstructural evolution during carbide precipitation in HEBM-synthesized CrMnFeNiCo-based HEAs. The objective is to improve our understanding of the precipitation conditions.

## 2. Materials and Methods

### 2.1. High-Energy Ball Milling

The raw materials employed were elemental powders of Co, Cr, Fe, Mn, Ni, Mo, and V (99.8%, Sigma-Aldrich, Toluca, Mexico State, Mexico). The CrMnFeCoNi, CrMnFeCoNiV_0.5_, and CrMnFeCoNiMo_0.5_ HEAs were synthesized through mechanical alloying using HEBM in a SPEX mill-8000M (Metuchen, NJ, USA). The milling conditions were set to: (i) 15 h of milling time, (ii) powder mass 8.5 g, (iii) ball-to-powder ratio of 5:1, (iv) argon atmosphere, and (v) 1 mL of heptane as PCA. The milling runs were conducted in cycles of 60 min of milling with 30 min pause, and forced ventilation was used to prevent overheating. The elemental analyses of the HEAs through ICP-OES are summarized in Table 1, which reveals carbon concentrations of 0.79–0.91 wt.%.

### 2.2. HEA Characterization

Differential thermal analysis (DTA) was performed to evaluate the thermal stability of the synthesized alloys. A PerkinElmer STA 6000 simultaneous thermal analyzer (PerkinElmer, Shelton, CT, USA) was used in a temperature range between 298 and 1173 K. The measurements were collected at 5, 10, 15, and 20 K/min heating rates with an N_2_ flow of 20 mL/min. For each DTA run, 10 ± 2 mg of sample was used. The reaction enthalpy (*ΔH_r_*) of each alloy was calculated using Pyris Basic Data Analysis software version 9.0.2.0193 (2008). Thermo-XRD runs were carried out on a Panalytical Philips X’Pert diffractometer (Philips, Amsterdam, The Netherlands) using Cu Kα1 radiation (λ = 1.5406 Å), over 20–100° in 2θ with a step size of 0.016°. The diffractometer is equipped with an Anton Paar HTK-1200N furnace (Anton Paar, Graz, Austria) for in situ heating (298–1473 K) and a TCU 2000/20 Temperature Control Unit (Shanghai Linbel Instrument Co., Ltd, Shanghai, China). To analyze the thermal stability of the alloys, thermo-XRD analyses were performed under vacuum with a heating rate of 10 K/min. The temperatures studied were selected based on DTA analyses of the CrMnFeCoNi, CrMnFeCoNiV_0.5_, and CrMnFeCoNiMo_0.5_ alloys, respectively. The XRD patterns were indexed using X’Pert High Core Plus software version 3.0e to identify the phases in the HEAs. The chemical composition of HEAs was determined using an inductively coupled plasma-optical emission spectrometer (ICP-OES) iCAP 7400 (Thermo Fisher Scientific, Waltham, MA, USA). A field emission scanning microscope (JSM7401F, JEOL, Tokyo, Japan), equipped with an EDS detector (EDAX, Pleasanton, CA, USA) was used to examine the cross-section of the alloyed powders obtained from the thermo-XRD experiments. Conventional metallographic techniques were used to prepare the alloyed powders on a conductive bakelite substrate. The samples were sanded to #2000 silicon carbide sandpaper and polished with 5 µm alumina powder.

### 2.3. Mathematical Models

The activation energy of carbide crystallization was analyzed using the Kissinger (Equation (1)) and Flynn/Wall/Ozawa (Equation (2)) models based on the ASTM E698 and ASTM E2890 standards, respectively [14,15]. These models relate the peak temperature and the heating rates through the following equations:

Kissinger’s model

(1) $$In\left(\frac{\beta }{T_{P}^{2}}\right)=\left(\frac{-E_{a}}{{RT}_{P}}\right)+k_{1}$$

Flynn/Wall/Ozawa’s model

(2) $$ln(\beta )=\left(\frac{-E_{a}}{{RT}_{P}}\right)+k_{2}$$

where *β* is the heating rate, *T_p_* is the peak temperature, *R* is the universal gas constant, and *k*_1_ and *k*_2_ are the kinetic constants. The Kissinger plot *ln(β/T_p_^2^)* vs. *1/T_p_* and Ozawa plot *ln(β)* vs. *1/T_p_* were built with *T_p_* and *β* data collected from the DTA curves. The slope of the plots was calculated using a linear regression statistical model. The activation energy (*E_a_*) was obtained from the relationship *E_a_ = R×slope*. In addition, the transformed fraction (*α*) of the carbide was obtained from the area under the DTA curve of the exothermic processes using the following equation (Equation (3)) [16]:

(3) $$\alpha =\frac{∆(A)}{A_{T}}$$

where Δ(*A*) is the area under the curve at a specific temperature, and *A_T_* is the total area under the curve. Kinetic parameters for carbide crystallization/formation were obtained by plotting the transformed fraction vs. temperature (*α* vs. Δ*T*). The curves were fitted using the Avrami–Erofeev model (Equation (4)) [17].

(4) $$f(\alpha )=1-e^{({-k_{c}∆T)}^{n}}$$

where *k_c_* is the reaction rate, Δ*T* is the reaction coordinate under non-isothermal conditions, and *n* indicates the grade of reaction. This model was selected for its ability to accurately represent precipitation processes involving random nucleation. Furthermore, the model simulates the evolution of precipitation over time and in response to temperature variations.

## 3. Results and Discussions

### 3.1. Characterization of Powders Obtained Through HEBM

**Figure 1:**
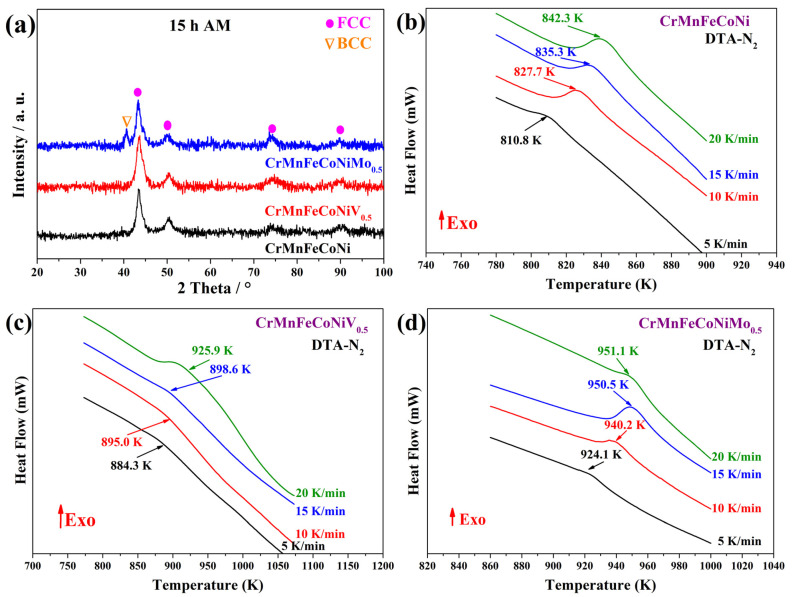
(**a**) XRD patterns of HEAs obtained through HEBM for 15 h and DTA curves of (**b**) CrMnFeCoNi, (**c**) CrMnFeCoNiV_0.5_, and (**d**) CrMnFeCoNiMo_0.5_ HEA powders.

XRD scans reveal the structure of HEA powders after 15 h of mechanical alloying through HEBM (Figure 1a). All patterns show broadened, shortened peaks after HEBM, indicating grain refinement and internal tension. Only the CrMnFeCoNiMo_0.5_ sample shows a slight shift toward lower angles, indicating a small variation in the lattice parameter.

The CrMnFeCoNi and CrMnFeCoNiV_0.5_ systems formed HEAs in solid solution with a face-centered cubic (FCC) structure. Meanwhile, the diffractogram of the CrMnFeCoNiMo_0.5_ HEA indicates the presence of a peak at 40.57° in 2θ, in addition to the peaks attributed to FCC phase signals. The new reflection was associated with a body-centered cubic structure (BCC) similar to that of Mo (ICSD-00-001-1205: cubic structure, *I m*$\bar{3}$*m*, *a* = 3.14 Å). Therefore, the CrMnFeCoNiMo_0.5_ HEA shows a mixture of FCC/BCC structures. However, there is no evidence of carbide crystallization in the HEAs, as has been reported in other studies [7].

On the other hand, thermograms obtained through DTA of CrMnFeCoNi, CrMnFeCoNiV_0.5_, and CrMnFeCoNiMo_0.5_ HEAs at different heating rates are shown in Figure 1b–d. The DTA curves of the HEAs showed a single peak between 298 and 1173 K, indicating heat release. Therefore, they were associated with an exothermic process. Furthermore, for each HEA, the peak shifted to higher temperatures as the heating rate increased, because a higher rate did not allow the process to reach completion at the temperature that would have been achieved at a lower rate [17]. The temperature peaks in CrMnFeCoNi, CrMnFeCoNiV_0.5_, and CrMnFeCoNiMo_0.5_ HEAs shift from 810.8 to 842.2 K, 884.2–925.9 K, and 924.0–951.1 K, respectively. A broad exothermic peak is observed in the DTA curves of the CrMnFeCoNiV_0.5_ HEA at heating rates of 5, 10, and 15 K/min, whereas at 20 °C/min the peak broadens further. This behavior makes it difficult to know exactly when the exothermic process begins and ends. As some authors have reported, M_x_C_y_-type carbide crystallization in steel and superalloys occurs at ~823 K [18,19]. Hence, the observed exothermic peaks correspond to the crystallization of M_x_C_y_-type carbides. It has also been reported that, at temperatures above 923 K, carbides undergo phase transformations [20]. This transformation is consistent with what was reported in the present work. In addition, the DTA results (Figure 1b–d) showed that the presence of Mo in the HEA promotes exothermic peaks at higher temperatures due to its high melting point.

### 3.2. Structural Evolution at High Temperatures

**Figure 2:**
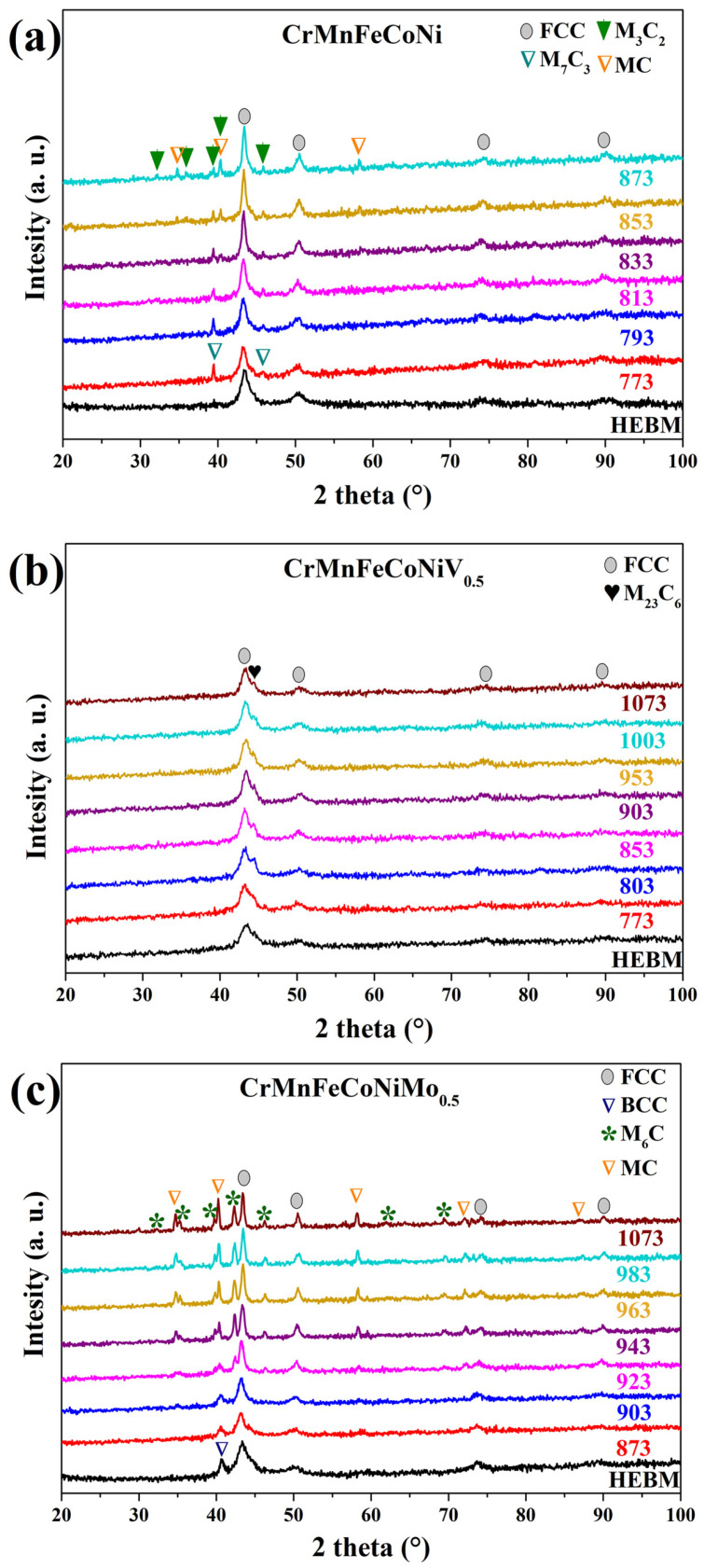
Thermo-XRD patterns of (**a**) CrMnFeCoNi, (**b**) CrMnFeCoNiV_0.5_, and (**c**) CrMnFeCoNiMo_0.5_ HEAs.

Thermo-XRD experiments confirmed that the carbide (M_x_C_y_) crystallized at the temperature where the DTA graphs showed an exothermic event (Figure 2). Typically, HEAs obtained through HEBM form multi-element carbides after a heat treatment [6,7]. Therefore, the term “M_x_C_y_” was used to denote carbides in this study, where “M” denotes the metal mixture and “C” denotes carbon. In the XRD patterns of the CrMnFeCoNi HEA (Figure 2a), the peaks associated with the FCC phase reveal a narrowing of the full-width half-maximum (FWHM) as the temperature increases. This phenomenon is associated with an increase in crystallinity. Additionally, two new peaks were observed at 773 K (39.4° and 45.9° in 2θ). According to the Inorganic Crystal Structures Database (ICSD), these peaks can be identified with an M_7_C_3_-type structure (ICSD-03-065-1347: orthorhombic structure, *Pnma*, *a* = 4.53 Å, *b* = 7.01 Å, *c* = 12.14 Å). An increase in temperature at 833 K resulted in the formation of new peaks that exhibited a structural similarity to the MC type (ICSD-00-010-0181: cubic structure, *F m*$\bar{3}$*m*, *a* = 4.47 Å). It can be observed that as the peak intensity of the MC-type structure increases, the peak intensity of the M_7_C_3_-type structure decreases. However, at 853 K, new reflections emerged, indicating the presence of an M_3_C_2_-type structure (ICSD-00-035-0804: orthorhombic structure, *Pnma*, *a* = 5.52 Å, *b* = 2.82 Å, *c* = 11.46 Å).

According to Ellingham’s diagrams, among the elements that comprise HEAs, Cr is the transition metal most likely to form carbides [21]. The order of chromium carbide crystallization is determined by their Gibbs free energy (Δ*G*); carbides with more negative values tend to form more easily. The Δ*G* values for Cr_7_C_3_, Cr_23_C_6_, and Cr_3_C_2_ are −158.3 kJ/mol, −89.5 kJ/mol, and −53.1 kJ/mol, respectively [22]. However, the CrC is a metastable phase that exists at high temperatures and has a positive Δ*G* value [23]. Therefore, based on the available literature and the observed results, it is suggested that Cr-rich nanocarbides form in the following order of increasing temperature: M_7_C_3_, M_3_C_2_/MC.

In Figure 2b, the XRD patterns of the alloy with V do not exhibit any signs of recrystallization of the FCC phase, which is discussed later. Furthermore, an additional reflection at 44.35° in 2θ was observed as the temperature increased. This peak was associated with a M_23_C_6_-type structure (ICSD-00-035-0783: cubic structure, *F m*$\bar{3}$*m*, *a* = 10.66 Å). The presence of M_23_C_6_-type carbide in the diffractogram of HEA obtained by HEBM is possible, although the signals could be hidden by the broadening of the peak at 43.46° in 2θ. Nevertheless, the peak lost intensity after 903 K. This behavior is related to the loss of crystallinity. The solubility of carbides has been observed during high-temperature annealing of steels [24]. Therefore, it can be argued that carbon from the M_23_C_6_-type structure is solubilized into the matrix at high temperatures. As the temperature increases in thermo-diffraction experiments, the diffractograms of CrMnFeCoNiMo_0.5_ HEA (Figure 2c) demonstrate a narrowing of the FWHM from peaks that correspond to the FCC phase. This narrowing suggests an increase in crystallinity. Furthermore, peaks associated with the MC-type structure were observed at 903 K. However, at 923 K, new peaks were observed. The new reflections coincide with an M_6_C-type structure (ICSD-00-049-1609: cubic structure, *F d*$\bar{3}$*m*, *a* = 11.10 Å). The signal from both new structures increases with increasing temperature. This phenomenon can be attributed to two factors: a higher degree of carbide crystallinity or an increase in the proportion of carbide phases within the HEAs. It has been reported that high-speed steels and superalloys with high Mo content often form carbides with an M*_6_*C-type structure and, in small quantities, metastable MC-type structure [25].

**Figure 3:**
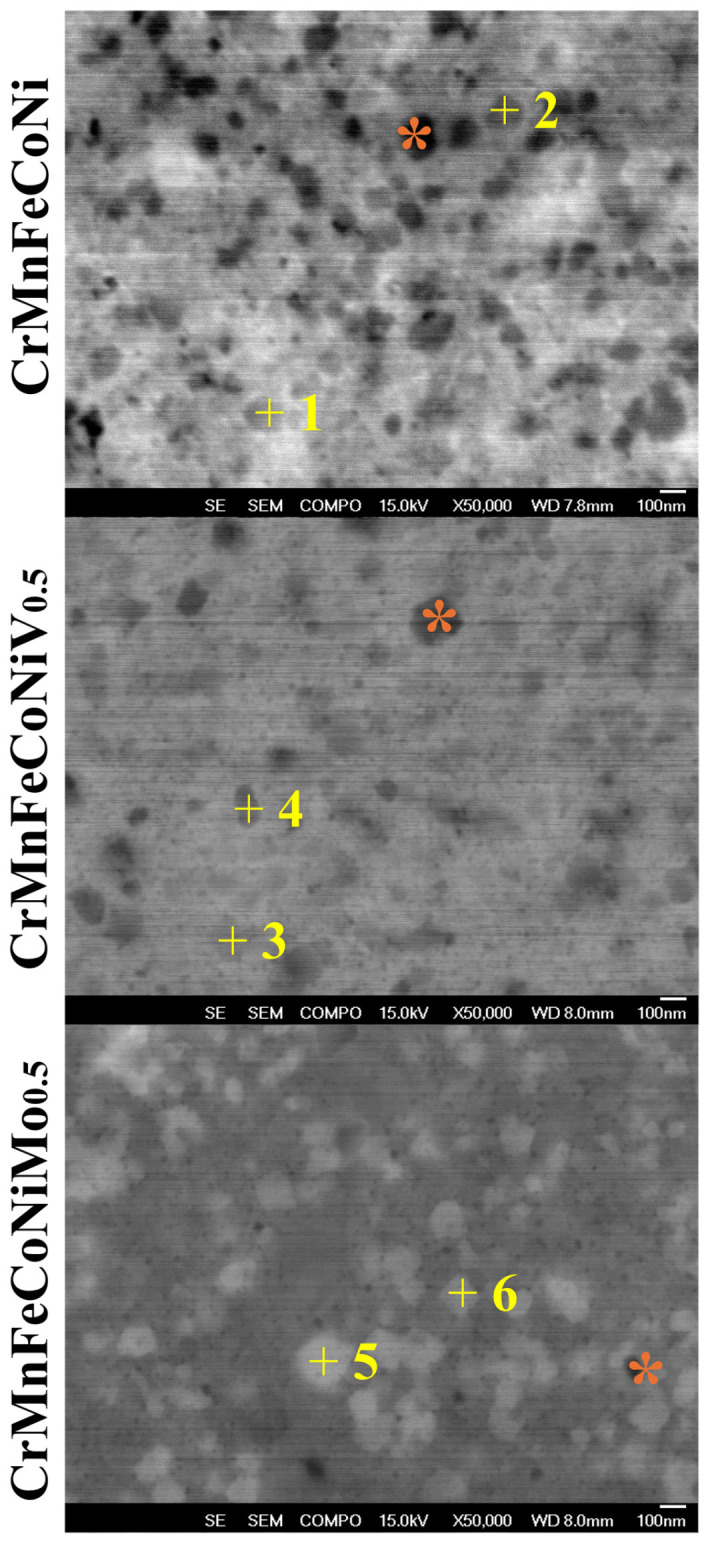
SEM micrographs of CrMnFeCoNi, CrMnFeCoNiV_0.5_, and CrMnFeCoNiMo_0.5_ HEAs.

The analysis of the cross-section of HEA powders through SEM-EDS revealed the formation of irregularly shaped nanoprecipitates with a diameter of 100 nm (Figure 3). The presence of dark-colored microstructures (orange asterisk) in all HEAs was associated with the formation of oxides, while microstructures with grayscale were associated with the presence of M_x_C_y_ nanophases. Furthermore, the use of grayscale in micrography facilitates the differentiation of regions associated with different phases. Therefore, the brighter or clearer regions in the micrograph indicate elements with higher electron density.

The elemental composition obtained through SEM-EDS is a semi-qualitative analysis; therefore, its findings should not be considered definitive. However, it does allow us to determine the predominant element in each phase (Table 2). The M_x_C_y_ phases in the CrMnFeCoNi and CrMnFeCoNiV_0.5_ HEAs revealed a high Cr content. Meanwhile, the CrMnFeCoNiMo_0.5_ HEA indicates that the carbides are rich in Mo and Cr. According to reports in the literature, 1 wt.% C in HEAs is sufficient to promote the crystallization of M_x_C_y_-type chromium nanocarbides [22]. In this study, although the HEA obtained through HEBM had lower C content, carbides were observed post-thermo-XRD experiments.

### 3.3. Effect of V and Mo on the Fundamental Thermodynamic Parameters and Kinetics of M_x_C_y_ Crystallization in HEAs

**Figure 4:**
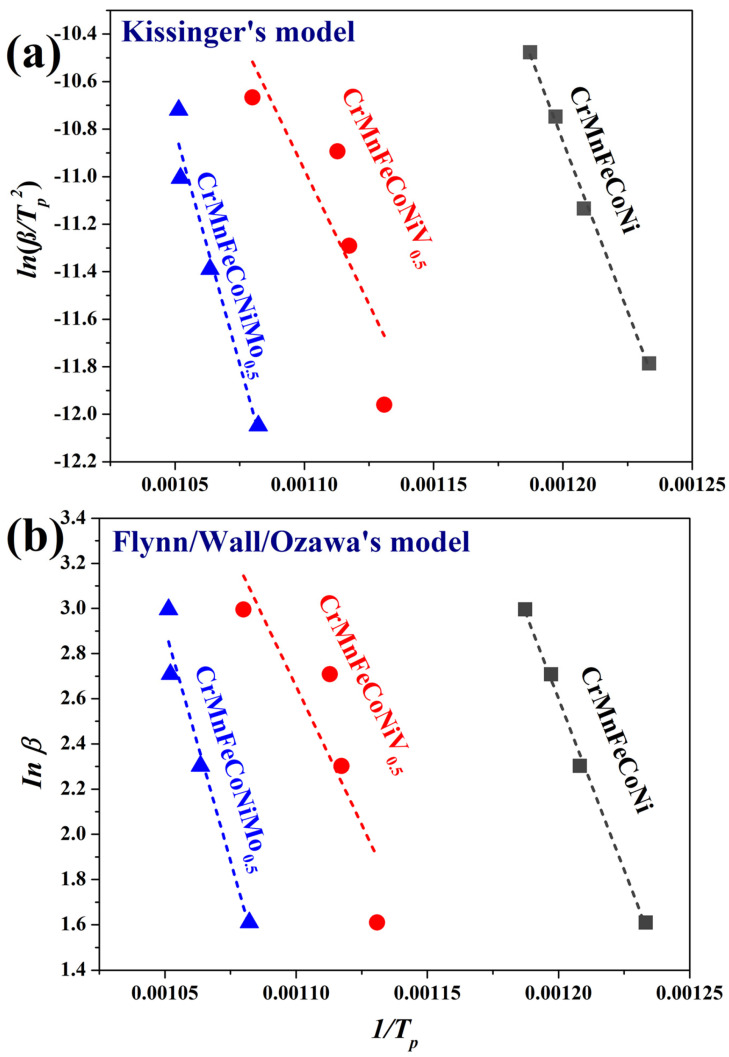
(**a**) *ln(β/*T*_p_^2^)* vs. *1/*T*_p_* and (**b**) *ln(β)* vs. *1/*T*_p_* plots of Kissinger and Flynn/Wall/Ozawa models, respectively.

From thermograms in HEAs after HEBM (Figure 1b–d), the heating rate (*β)* and peak temperature (*T_p_*) were obtained, and from these data, the Kissinger and Flynn/Wall/Ozawa models were plotted (Figure 4). The activation energy (*E_a_*) for carbide crystallization was calculated with the slope of the linear regression. All the values are summarized in Table 3. A high *E_a_* value indicates that a large amount of energy is required for a thermal reaction. In addition, the CrMnFeCoNiV_0.5_ HEA exhibits a higher standard deviation in *E_a_*. It has been reported that variations in the *E_a_* of carbide precipitation in superalloys are attributed to the predominance of diffusion of a specific element, which acts as a controlling factor in the precipitation process [26]. Therefore, it could be suggested that the diffusion of V in the HEA affects the pre-precipitation process. The results of the thermo-XRD patterns analyses (Figure 2) indicate that the peaks of the DTA curves for the CrMnFeCoNi, CrMnFeCoNiV_0.5_, and CrMnFeCoNiMo_0.5_ HEA powders are associated with the following carbide crystallization sequences: M_7_C_3_→MC→M_3_C_2_, M_23_C_6_, and MC→M_6_C, respectively. The carbides formed in the CrMnFeCoNi HEA were found to be composed primarily of Cr. Moreover, the incorporation of V into HEA promotes the crystallization of Cr-rich M_23_C_6_ through diffusion mechanisms that slow down the kinetics. In the case of HEA with Mo, the carbides were found to be rich in Mo. The MC-type carbide is a metastable phase observed at elevated temperatures and serves as a precursor to the more stable M_6_C-type phase.

Pearson’s correlation coefficients (*r*) for the linear regressions of the Kissinger and Flynn/Wall/Ozawa models were similar across all HEAs. However, *r* was >0.98 for the CrMnFeCoNi and CrMnFeCoNiMo_0.5_ HEAs, while for the CrMnFeCoNiV_0.5_ HEA it was ~0.87. The low value of *r* is due to a broadening exothermic peak, which makes it difficult to correctly identify the T*_p_* (Figure 1c). Saumitra, V. (2025) [26] studied the precipitation kinetics of carbides in an Inconel 718 alloy using the Johnson–Mehl–Avrami–Kolmogorov (JMAK) equation. The model fit values were *r* = 0.9 and *r*^2^ = 0.81. The low fit value was associated with a disparity in *E_a_*, attributed to the predominance of Nb diffusion as the controlling factor in carbide precipitation [26]. Therefore, V diffusion processes in CrMnFeCoNi HEA are key factors in carbide precipitation inside the alloy. In contrast, Mo diffusion does not impact the *E_a_* of carbide precipitation processes.

The kinetic constants *k*_1_ and *k*_2_ are influenced by the slope of the linear regression; a higher slope indicates a greater energy requirement for crystallization and a higher energy input to reach the reaction’s peak temperature. Furthermore, the average of the reaction enthalpy (Δ*H_r_*) obtained at different temperatures from the DTA curves (Figure 1b–d) using Pyris Basic Data Analysis software version 9.0.2.0193 (2008) for carbide crystallization showed the following trend: CrMnFeCoNi (−44.3 J/g), CrMnFeCoNiMo_0.5_ (−93.8 J/g), and CrMnFeCoNiV_0.5_ (−223.8 J/g) HEAs.

Oppermann et al. [27] analyzed the carbide crystallization behavior in a 10Co-10Cr-2.5 V-1.5C medium-entropy alloy (MEA) and reported a slowdown in carbide crystallization kinetics compared to other FCC MEAs and austenitic steels. This phenomenon is related to the slower diffusion of V due to the high plastic deformation and its intrinsic diffusion coefficient. Wang et al. [28] have investigated the influence of Mo on the thermodynamic behavior of carbide crystallization in steels. They reported that Mo favors the nucleation of carbides in steel and superalloys from a Gibbs free energy perspective [28,29].

**Figure 5:**
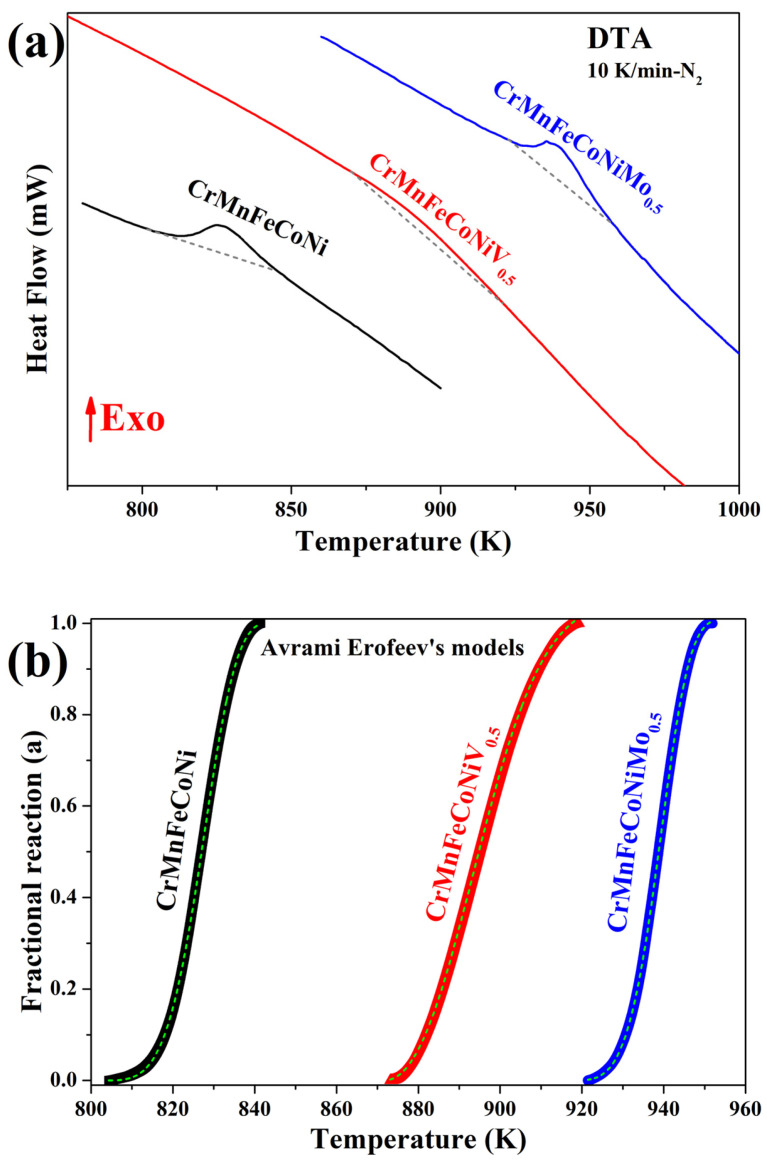
(**a**) DTA curves and (**b**) transformed fraction (*α*) of CrMnFeCoNi, CrMnFeCoNiV_0.5_, and CrMnFeCoNiMo_0.5_ HEA powders.

The themograms with 10 K/min heating rate were selected to evaluate the transformed fraction (*α*) and crystallization kinetics (Figure 5a). A dashed line in the region of the exothermic peak was drawn to indicate the start and end of the crystallization process. The *α* vs. *T* plots show sigmoid behavior (Figure 5b), indicating that the nucleation-growth mechanism controls the transformation. The Avrami–Erofeev model was used to fit the curves and obtain the kinetic parameters for carbide crystallization. The kinetic constant (*k_c_*) and grade of reaction (*n*) values are summarized in Table 4. The coefficient of determination (*r*^2^) is a measure of how well the model reproduces the observed outcomes. It can be observed that the Avrami–Erofeev model fits the HEA experimental data perfectly (*r*^2^ = 0.998). The *k_c_* values are comparable among HEAs. However, HEAs with Mo exhibit slightly higher values, suggesting a faster transformation rate. The *n* values of HEAs revealed the following trend: CrMnFeCoNiV_0.5_ (2.319), CrMnFeCoNi (4.485), and CrMnFeCoNiMo_0.5_ (4.5121). Low values of *n* correspond to a phase transformation driven by diffusion mechanisms, whereas high values indicate that the crystallization/transformation is controlled by nucleation at pre-existing nucleation sites [14]. In this work, all *n* values are >1, confirming that carbide crystallization is controlled by nucleation. From Figure 2c, it is observed that V slows down the crystallization kinetics. In addition, the kinetic has a lower *n* value than other HEAs. Therefore, it can be inferred that V in HEA maintains stable structural defects in the FCC matrix, as observed in the thermo*-*XRD experiments (Figure 2c). This behavior can promote slow diffusion during carbide growth and slow recrystallization of the FCC matrix.

## 4. Conclusions

This study presents the sequence and kinetics of crystallization of M_x_C_y_-type nanocarbides in HEAs manufactured via powder metallurgy. Additionally, the effects of V and Mo on carbide crystallization were examined. The results revealed that a concentration between 0.6 and 1.0 wt.% C is trapped during the HEBM process, and it is thermodynamically sufficient to form carbides in the HEAs. Thermo-XRD experiments demonstrated distinct carbide crystallization sequences at elevated temperatures for CrMnFeCoNi (M_7_C_3_→MC→M_3_C_2_), CrMnFeCoNiV_0.5_ (M_23_C_6_), and CrMnFeCoNiMo_0.5_ (MC→M_6_C). On the other hand, the results indicated that the phase transitions in the DTA curves of CrMnFeCoNi, CrMnFeCoNiV_0.5_, and CrMnFeCoNiMo_0.5_ are associated with the crystallization of the M_7_C_3_, M_23_C_6_, and MC phases, respectively. Furthermore, the Δ*H_r_* values for M_7_C_3_, M_23_C_6_, and MC were −44.3, −93.8, and −223.8 J/g, while the *E_a_* values were 238–251, 188–203, and 326–341 kJ/mol, respectively. On the other hand, the kinetic parameters indicate that the crystallization of M_x_C_y_-type nanocarbides is controlled by nucleation.

The incorporation of V into HEA retards the recrystallization of the HEA FCC matrix and slows down the crystallization kinetics of M_23_C_6_. Finally, Mo in HEA facilitates the crystallization of a metastable MC type, which serves as a precursor for the subsequent crystallization of a more stable M_6_C type. In summary, the nanocarbide crystallization behavior during the conventional sintering of the modified HEA is very similar to that reported for steels and superalloys. This knowledge will help design HEAs with controlled carbide microstructures, enabling fine-tuning of the performance of engineering components.

## Figures and Tables

**Table 1:** Elemental analysis of HEAs through ICP-OES.

Sample	Elemental Analysis/wt.%								
	Cr	Co	Mn	Fe	Ni	V	Mo	C	O
High-Energy Ball Milling 15 h									
CoCrFeMnNi	18.28	22.39	18.75	18.48	21.29		----	0.79	0.01
CoCrFeMnNiV_0.5_	16.33	19.78	17.26	17.02	21.03	7.68	----	0.89	0.01
CoCrFeMnNiMo_0.5_	15.42	18.70	16.29	16.14	17.66	----	14.88	0.91	0.01

**Table 2:** Semi-quantitative elemental analysis of HEAs post-thermo-XRD experiments through SEM-EDS in Figure 3.

Scan Points	Elemental Analyses/wt.%								
	Cr	Co	Mn	Fe	Ni	V	Mo	C	O
1	22.7	16.8	15.7	16.7	15.6			9.8	2.6
2	19.5	17.5	16.3	17.7	16.4			9.9	2.3
3	18.5	16.3	14.0	13.5	16.0	7.1		9.5	1.7
4	21.3	13.2	12.4	14.2	12.9	8.0		15.3	2.6
5	11.7	9.1	7.3	15.5	6.4		41.0	11.2	2.0
6	16.8	13.3	13.9	14.2	14.2		9.1	16.9	1.6

SEM-EDS analysis reveals a semi-quantitative elemental composition of each phase.

**Table 3:** *β, T_p_, E_a_*, and Δ*H_r_* data collected from DTA curves and Kissinger and Flynn/Wall/Ozawa models (Figure 1 and Figure 2).

HEA	*β* K/min	*T_p_* K	Kissinger’s Model kJ/mol	Ozawa’s Model kJ/mol	Δ*H_r_* J/g
CoCrFeMnNi	5	810.84	*r* = 0.998 *E_a_* = 238.2 ± 10.1 *k*_1_ = 23.5 ± 1.5	*r* = 0.998 *E_a_* = 251.9 ± 20.2 *k*_2_ = 38.9 ± 1.5	−51.223
	10	827.73			−34.631
	15	835.32			−33.192
	20	842.26			−58.234
CoCrFeMnNiV_0.5_	5	884.26	*r* = 0.863 *E_a_* = 188.3 ± 78.0 *k*_1_ = 13.9 ± 10.4	*r* = 0.879 *E_a_* = 203.3 ± 77.9 *k*_2_ = 29.6 ± 10.4	−267.606
	10	895.01			−176.531
	15	898.60			−120.666
	20	925.97			−330.536
CoCrFeMnNiMo_0.5_	5	924.07	*r* = 0.981 *E_a_* = 326.2 *k*_1_ = 30.4 ± 5.8	*r* = 0.983 *E_a_* = 341.8 *k*_2_ = 46.1 ± 5.8	−96.238
	10	940.25			−80.423
	15	950.49			−104.746
	20	951.13			−29.204

**Table 4:** Kinetic parameters (*k****_c_*** and *n*) of the Avrami–Erofeev model for the crystallization/transformation.

HEA	*k_c_*	*n*	*r* ^2^
CoCrFeMnNi	0.0348	4.485	0.999
CoCrFeMnNiV_0.5_	0.0350	2.319	0.999
CoCrFeMnNiMo_0.5_	0.0389	4.521	0.999

*r*^2^ is a measure of the quality of the model fit.

## Data Availability

Data will be made available on request.
